# Postnatal Development of Visual Cortical Function in the Mammalian Brain

**DOI:** 10.3389/fnsys.2020.00029

**Published:** 2020-06-09

**Authors:** Chand Parvez Danka Mohammed, Reem Khalil

**Affiliations:** ^1^Biosciences and Bioengineering Research Institute (BBRI), American University of Sharjah, Sharjah, United Arab Emirates; ^2^Department of Biology, Chemistry, and Environmental Sciences, American University of Sharjah, Sharjah, United Arab Emirates

**Keywords:** visual function, brain development, refinement, maturation, ferret, monkey, human

## Abstract

This review aims to discuss (1) the refinement of mammalian visual cortical circuits and the maturation of visual functions they subserve in primary visual cortex (V1) and other visual cortical areas, and (2) existing evidence supporting the notion of differential rates of maturation of visual functions in different species. It is well known that different visual functions and their underlying circuitry mature and attain adultlike characteristics at different stages in postnatal development with varying growth rates. The developmental timecourse and duration of refinement varies significantly both in V1 of various species and among different visual cortical areas; while basic visual functions like spatial acuity mature earlier requiring less time, higher form perception such as contour integration is more complex and requires longer postnatal time to refine. This review will highlight the importance of systematic comparative analysis of the differential rates of refinement of visual circuitry and function as that may help reveal underlying key mechanisms necessary for healthy visual development during infancy and adulthood. This type of approach will help future studies to establish direct links between various developmental aspects of different visual cortical areas in both human and animal models; thus enhancing our understanding of vision related neurological disorders and their potential therapeutic remedies.

## Introduction

Cortico-cortical networks undergo significant refinement and reorganization in the postnatal period; humans (Huttenlocher, 1979; Burkhalter et al., 1993), monkeys (Barone et al., 1995; Coogan and Van Essen, 1996; Batardière et al., 2002), cats (Price and Blakemore, 1985; Price, 1986; Dehay et al., 1988; Price and Zumbroich, 1989; Batardiere et al., 1998), ferrets (Durack and Katz, 1996; Ruthazer and Stryker, 1996). Similarly, neuronal response properties in V1 and other visual cortical areas mature throughout development (Bienenstock et al., 1982; Frégnac and Imbert, 1984; Zhang et al., 2005). As a result, visual functions also mature throughout the developmental period (El-Shamayleh et al., 2010; Braddick and Atkinson, 2011). Although the developmental sequence of when distinct visual functions emerge, when the critical period (CP) for damage is, and when functions are adultlike are of great interest to developmental neuroscientists, they are not completely understood.

Neural circuit formation and functional maturation in the developing brain follows a pattern of either hierarchical maturation (Flechsig, 1901; Bourne and Rosa, 2006; Tierney and Nelson, 2009; Okazawa et al., 2016) or synchronous maturation (Law et al., 1988; Chapman et al., 1996; Rao et al., 1997; Khalil and Levitt, 2013, 2014). Some aspects of cortical circuitry, receptive field (RF) physiology, and visual functions mature in a hierarchical fashion; humans (Tierney and Nelson, 2009; Brown and Jernigan, 2012), monkeys (Zhang et al., 2005, 2008; Zheng et al., 2007), while others develop in a synchronous manner; ferrets (Khalil and Levitt, 2013, 2014). Furthermore, the developmental timeline of different visual functions varies significantly among visual cortical areas within a species (Sur and Leamey, 2001; Hooks and Chen, 2007; Empie et al., 2015; Atkinson, 2017), and across species (Harwerth et al., 1986, 1990; Lewis and Maurer, 2005; Braddick and Atkinson, 2011). The timeline for neural events in the cortex of a P0 ferret is equivalent to gestational days G80 in macaque and G104 in humans (Clancy et al., 2001). Normalization of different developmental trajectories among species does not seem to eliminate the difference in the rate of maturation of different visual functions (Finlay and Darlington, 1995; Clancy et al., 2001; Finlay et al., 2001, 2011; Nagarajan et al., 2010; Marchetto et al., 2019). Therefore, the fact that different functions mature at different rates may be attributed to species differences and how different brain regions and their underlying mechanisms depend on visual experience.

Although other reviews have focused on the development of (1) Ocular dominance columns in ferrets, cats and monkeys (Katz and Crowley, 2002), (2) RF properties of neurons in mice and ferrets (Huberman et al., 2008), (3) Visual cortical circuits in cats (Katz and Callaway, 1992), and (4) Human visual function (Braddick and Atkinson, 2011), the present review comprehensively discusses the overall development and maturation of visual cortical circuits underlying function and behavior along with their differential rates of refinement in different mammalian species. We limit the scope of this discussion to human, monkey, and ferret studies for the following reasons. We emphasize human behavioral studies as great efforts have been made to understand the normal development of visual function as well as potential for visual dysfunction. Moreover, anatomical and functional development of the visual cortex has traditionally been studied in monkeys which has led to a large body of literature. Similarly, the ferret has emerged as a model organism in current visual developmental studies due to a number of advantages that we discuss in this review. Rodent models will not be extensively discussed as they are primarily used to reveal molecular mechanisms of visual developmental events (see reviews by Huberman et al., 2008; Feldheim and O’Leary, 2010; Espinosa and Stryker, 2012). The essential aspects of this discussion will be summarized under the following sections: (1) Development of visual cortical connections (2) Development of neuronal response properties in visual cortex, and (3) Maturation of visual function.

### Development of Visual Cortical Connections

Developmental remodeling of visual cortical circuits spans over successive stages of postnatal development in various species, (1) Corticocortical connections in ferrets (Durack and Katz, 1996; Ruthazer and Stryker, 1996; Khalil and Levitt, 2013, 2014); humans (Huttenlocher, 1979; Burkhalter et al., 1993) (2) Intracortical connections in cats (Anderson et al., 1988; Ghose et al., 1994; Horton and Hocking, 1996); monkeys (Lund and Harper, 1991; Lund and Holbach, 1991; Lund et al., 1991), and (3) Interhemispheric circuits in rats (Olavarria and Van Sluyters, 1985); hamsters (Norris and Kalil, 1992). Different anatomical features of visual cortical circuits are modified throughout development. For instance, axon and bouton density of feedforward projections from ferret area 17 to multiple cortical targets decline during the period after eye opening (Khalil et al., 2018). Similarly, the laminar distribution of feedback projections in the visual cortex of infant monkeys changes in the first 2 months of life, due to a decrease in the proportion of supragranular neurons (Barone et al., 1995). Importantly, the developmental refinement of cortical circuits is thought to underlie the maturation of visual function and behavior.

#### Ferret as a Model for Visual Cortical Development

**FIGURE 1:**
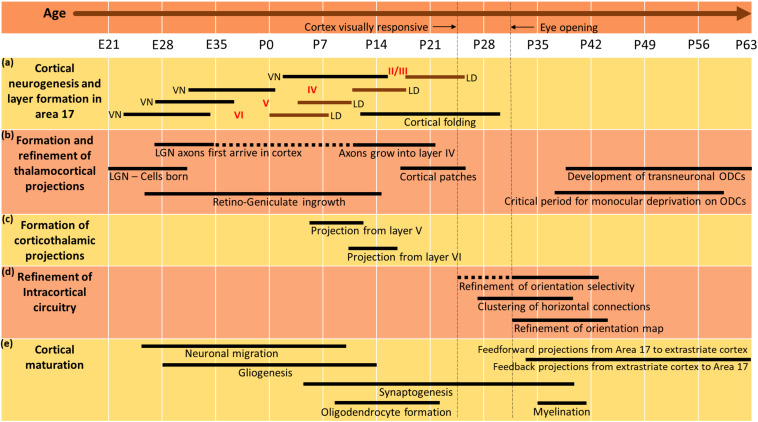
Timeline depicting major developmental milestones in the ferret visual cortex. The timeline of different developmental events in the ferret visual system, subdivided into five parts: **(a)** cortical neurogenesis and layer formation in area 17. The timing, progression and duration of neurogenesis and laminar differentiation are represented by horizontal bars, where VN and LD denote ventricular neurogenesis and laminar differentiation, respectively. Embryonic day 22 (E22) marks the earliest occurrence of neurogenesis in layer VI followed by other cortical layers over the next few weeks. In cortical layers II/III neurogenesis is not completed until postnatal day 14 (P14) and cortical folding is not complete until about postnatal day 30 (P30); **(b)** Formation and refinement of thalamocortical projections. LGN cells are born by E21 and axons first arrive in the visual cortex by E27. Ingrowth into layer IV occurs around P10 with the developmental projections of retino-geniculate fibers appearing as early as E27 and lasting until P14. Cortical patches are visible as early as P16. Formation of ocular dominance columns takes place around P37 and CP begins approximately around P35 (∼75 days after conception); **(c)** Formation of corticothalamic projections. Early projections from the cortex to the thalamus are formed by neurons in cortical layer V soon after birth, and subsequently followed by neurons in layer VI a week later; **(d)** Refinement of intracortical circuitry. At the onset of visual responsiveness in the cortex, one third of neurons show orientation selective responses, and the mature adult like pattern is not attained until around P45. Orientation map is formed by around P31 and clustered horizontal connections appear as early as around P27; **(e)** Cortical maturation. Developmental timecourse for several critical events such as neuronal migration, gliogenesis, synaptogenesis, oligodendrocyte formation and myelination in corticogenesis. The refinement of feedforward projections from area 17 to extrastriate cortex, and feedback projections from extrastriate areas to area 17 begins around P35, which is soon after eye opening (P30). Adapted from “Development and plasticity of cortical areas and networks” by Sur and Leamey (2001), Nature Reviews Neuroscience, 2(4), page 252. Copyright 2001 by Springer Nature.

The ferret has emerged as a promising model system in the study and characterization of visual circuit and function, particularly for early developmental mechanisms. This can be mainly attributed to the fact that ferrets are born at an early stage of brain development with delayed eye opening, which does not occur until about postnatal day 30 (Linden et al., 1981; Walsh and Guillery, 1985). The period after eye opening is critical for sculpting visual cortical circuits as the onset of visual experience is thought to play a major role in this process (Khalil and Levitt, 2013, 2014; Khalil et al., 2018). They have a sensory system which is similar to that of the cat, but being born developmentally earlier like mice and rats, many events critical in shaping cell-circuit assemblies, synaptogenesis, axonal targeting, laminar and sub-laminar specialization of neurons, and remodeling of cortico-cortical circuits take place postnatally (Jackson et al., 1989; Wong, 1999; Katz and Crowley, 2002; Reillo and Borrell, 2012). Figure 1 depicts major developmental milestones in the ferret visual cortex. Early events such as timing of cortical neurogenesis and layer formation in area 17 (Jackson et al., 1989), as well formation and refinement of thalamocortical projections are well documented (Johnson and Casagrande, 1993). Similarly, late events that ensue after eye-opening and depend on visual experience, include the refinement of horizontal projections in area 17 (Ruthazer and Stryker, 1996), as well as remodeling of corticocortical feedforward (Khalil et al., 2018) and feedback circuits in ferret visual cortex (Khalil and Levitt, 2014).

In addition to being important for studying developmental events, ferrets are also useful subjects for comparative studies. Comparative studies can reveal which physiological or anatomical aspects are present in all mammals, and which are unique to a particular species, For instance, it has been shown that the arrangement of ocular dominance domains in the ferret visual cortex exhibits a high degree of regional variation in size and shape, compared to that in primates and cats (Anderson et al., 1988; Horton and Hocking, 1996; White et al., 1999). This has been shown to result from the asymmetries in the crossed and uncrossed retinal pathways. Importantly, the late date of eye opening provides a long window of investigative access to the changes in emerging brain functions due to the onset of visual experience.

#### Patterns of Visual Cortical Development (Hierarchical Versus Synchronous)

Neural circuits underlying the maturation of visual functions are programmed to follow a hierarchical or synchronous pattern of maturation that is species dependent. While hierarchical maturation refers to the sequential development of basic physiological properties or anatomical circuits followed by the maturation of complex ones (Tierney and Nelson, 2009; Okazawa et al., 2016), synchronous maturation involves concurrent development of a given functional or anatomical aspect in multiple cortical areas (Law et al., 1988; Khalil and Levitt, 2013, 2014). To clarify why certain aspects of visual cortical circuits mature hierarchically and others develop in a synchronous manner, it is important to understand the nature of the interaction among various visual areas and the continuously changing connectivity patterns both within and among species during early postnatal development. Depending on the complexity of the visual function and the progressive interaction between multiple visual areas, neural circuits may prefer to adapt to one maturation pattern over the other. This preference of neural circuits may largely depend on feedforward and feedback mechanisms that facilitate the formation and rearrangement of visual pathways underlying function. The adaptation of neural circuits to a specific maturation pattern might have gradually evolved in different visual areas and various species over time.

One of the earliest reports supporting the notion of hierarchical development revealed that cortical connections linking somatosensory, primary motor, auditory and visual cortical regions were the very first to undergo myelination during the early stages of postnatal life in humans (Flechsig, 1901). Similarly, primate studies have suggested that feedforward and feedback connections may not develop and refine at the same rate. For instance, in macaques, there are clear differences in the rate of refinement of the laminar organization of corticocortical projecting neurons from V1 to V4 that are fully developed to adultlike level prenatally, whereas feedback pathways from V4 to V2 undergo extensive reorganization (Batardière et al., 2002). Also, in macaques, the laminar distribution of feedforward neurons from V1 to V2 is essentially adultlike during the early postnatal weeks of life, with further refinement of these projections occurring between 1 to 2 months postnatal (Baldwin et al., 2012). It seems that in human infants, feedforward pathways also refine earlier than feedback pathways (Burkhalter, 1993; Burkhalter et al., 1993). Therefore, the fact that the developmental refinement of feedforward pathways occurs before that of feedback pathways in humans and non-human primates suggests that it is an evolutionarily conserved mechanism which is adaptive in these highly visual species.

An open question remains as to why it is adaptive for feedforward connections to mature earlier than feedback connections. At the outset, feedforward and feedback connections in the visual cortex show strong differences in their anatomical features and connectivity patterns (Markov et al., 2014). Furthermore, two major claims have been made in this respect. Firstly, it is contended that feedforward connections are arranged in a topological fashion, whereas feedback connections are more diffusely organized both in terms of spatial extent of parent neurons along with their terminals and the frequency at which axonal bifurcation occurs. Secondly, feedback pathways are large in number and follow a path that crosses more hierarchical levels than feedforward pathways (Markov et al., 2014). Differences in the organizational principles and structural regularities of feedforward and feedback pathways result in physiological differences whereby feedforward signals generate RF properties, and feedback signals provide a modulatory influence (Hupé et al., 1998; Ekstrom et al., 2008). Furthermore, it is suggested that activation of feedforward pathways can give rise to quick and spontaneous characterization with little perceptual detail, whereas features of visual perception are provided by repetitive engagement of feedback connections (Hupé et al., 1998; Ekstrom et al., 2008; Markov et al., 2014). These functional differences between feedforward and feedback pathways are thought to reflect interactions that occur between prediction errors ascending the hierarchy and predictions descending the hierarchy, contributing to the differential rate of refinement of neural circuits (Markov et al., 2014). Previous reports also suggest that feedforward and feedback pathways are highly segregated and not restricted to the supragranular or infragranular layers, respectively (Barone et al., 2000; Markov et al., 2014). However, differences in the proportion of the parent cell bodies of feedforward and feedback pathways in the supragranular and infragranular layers may be another contributing factor leading to early adaptive maturation of feedforward pathways (Barone et al., 2000). Other evidence supporting the notion of hierarchical development of visual cortical circuits showed that V1 and area MT in monkeys refine earlier in time than other extrastriate areas, whereby V1 significantly influences the maturation of the dorsal stream and area MT supports ventral stream development (Mundinano et al., 2015). Early maturation and refinement of motion sensitive visual areas in monkeys highlights the important role of motion detection in the development of normal vision.

In contrast, there are reports which demonstrate concurrent development in the visual cortex. For instance, in ferrets, feedback neurons projecting from extrastriate areas to area 17 are already present prior to eye opening and refinement of the topography of feedback projections in these visual cortical areas occur in a synchronous manner (Khalil and Levitt, 2014). Additional evidence in ferrets reveals that multiple aspects of feedforward projections from area 17 to extrastriate visual areas refines at a similar rate (Khalil et al., 2018). The authors reported a steady decline in bouton density of feedforward projecting neurons from area 17 to areas 18, 19, and 21 from 4–8 weeks postnatally, suggesting a mechanism where feedforward circuits linking V1 to extrastriate areas reorganizes in a synchronous fashion. One possible explanation for the synchronous refinement of interareal circuits in the ferret visual cortex could be the delayed eye opening in this altricial species which is around P30 (Linden et al., 1981; Walsh and Guillery, 1985). This may cause the maturation of these cortical circuits in ferrets to be largely dependent on intrinsic activity, whereas plasticity dependent mechanisms driven by visual activity emerge shortly afterward (Ruthazer and Stryker, 1996; Sengpiel and Kind, 2002; Roy et al., 2018, 2020). Furthermore, this pattern of refinement may also be functionally crucial for the concurrent establishment of RF properties in multiple cortical areas mediated via feedforward projections, and the integration of inputs carried out by feedback circuits. Interestingly, the early development of feedforward projections in kittens seems to follow a hierarchical scheme whereby axon terminals of feedforward projecting neurons from area 17 are found first in area 18 and then in other extrastriate areas (Price and Zumbroich, 1989). In contrast, physiological studies in kittens have shown that the development of RF properties of neurons in area 17 and other extrastriate areas develop in parallel (Price et al., 1988). Therefore, it seems that although some aspects of feedforward circuits from area 17 to extrastriate cortex in cats develop in a hierarchal manner, RF properties of neurons across multiple visual areas can in fact develop concurrently. Given the shared similarities in the visual system of cats and ferrets, it would be intriguing to reveal whether the refinement of receptive field properties in multiple visual cortical areas of the ferret likewise ensue in a synchronous manner.

#### Developmental Refinement of Local Circuitry

A conspicuous feature of the neocortex is the horizontal arrangement of neurons within cortical areas (da Costa and Martin, 2010). Horizontal connections in primary visual cortex of carnivores such as cats and ferrets preferentially link neurons in cortical columns that have similar orientation preference (Hirsch and Gilbert, 1991; Ruthazer and Stryker, 1996; Bosking et al., 1997). Different anatomical features of horizontal connections have been shown to change throughout development. For instance, in cat area 17, the axon collaterals of horizontally projecting layer 2/3 and layer 5 pyramidal neurons reorganize during early stages of development (Callaway and Katz, 1990). Similarly, remodeling of axon collaterals of horizontal projections in ferret area 17 follows a similar developmental pattern to that seen in the cat (Durack and Katz, 1996) whereby patchy regions of axon branches first appeared by P34 followed by adultlike cluster formation seen at P45 (Durack and Katz, 1996; Sur and Leamey, 2001). Moreover, developmental changes of clustered connections occur synchronously with the maturation of orientation-selective responses (Chapman and Stryker, 1993). Previous experiments directly relating patterns of excitatory synaptic connectivity to visual response properties in mice V1 have revealed that the reorganization of intracortical connections between layer 2/3 excitatory neurons occur after the initial establishment of feedforward inputs from layer IV or the visual thalamus (Ko et al., 2013). Furthermore, the authors suggest that synaptic refinement in mouse V1 is due to the elimination and formation of connections, as well as changes in the strength of existing neural connections. The anatomical specificity of long-range horizontal connections in ferrets results through an activity-dependent process (Ruthazer and Stryker, 1996). Spontaneous activity within the cortical network serves in the initial establishment of these connections which later undergoes fine tuning. Additionally, horizontal connections have been shown to modulate long-range excitatory and inhibitory synaptic interactions between orientation columns in ferret area 17 (Weliky et al., 1995).

#### Development of Neuronal Response Properties in Visual Cortex

In the adult brain, neurons in the visual cortex respond to stimuli in a circumscribed region in visual space referred to as the receptive field center (RF). Visual stimuli presented in the surrounding region (surround) do not elicit a response, but can suppress or facilitate a cell’s response to simultaneous presentation of visual stimuli in its RF center (Hubel and Wiesel, 1965; Maffei and Fiorentini, 1976; Levitt and Lund, 1997; Sengpiel et al., 1997; Angelucci et al., 2002, 2017; Nurminen et al., 2018). The distinction between the receptive field center and surround allows researchers to understand how the information from multiple stimuli is encoded in the responses of individual neurons (Fitzpatrick, 2000). Consequently, center–surround interactions could potentially explain the phenomenon in which context modifies stimulus detectability. For instance, inhibitory effects are thought to be the basis for perceptual “pop-out”, curvature detection, and illusory contours (von der Heydt and Peterhans, 1989; Knierim and Van Essen, 1992; Lamme, 1995). Facilitatory surround effects have been linked to the processing of contour integration (Field et al., 1993; Kapadia et al., 1995). Cortical circuits implicated in mediating these contextual effects include long-range horizontal connections (Gilbert et al., 1996), and feedback projections arising from higher order areas that terminate in area 17 (Angelucci et al., 2002). Crucially, horizontal and feedback circuits that underlie these contextual effects contribute to global integration of visual signals.

The emergence of mature RF tuning properties during development is thought to arise from the refinement of cortical circuits that underlie these tuning properties. Converging evidence suggests that the developmental timecourse varies markedly for different response properties of visual cortical neurons. Moreover, the variability in development is reflective of the maturational state of cortical circuits that presumably mediate specific neuronal response properties. The functional maturation of the mammalian visual system appears to be slower in higher-order visual areas than in V1. Moreover, the maturation of neuronal response properties in the visual cortex is dependent on the maturation of cells at lower levels of the visual system (Boothe et al., 1988; Daw, 2005). The maturation of RF tuning properties during development underlies the improvement in visual performance by higher order mammals like humans and monkeys (Tanaka and Fujita, 2000; Daw, 2005). Previous findings have shown that the maturity of RF properties of neurons can partially account for the improvement of behavioral performance in macaques (Kiorpes et al., 2003). Neural processing of photoreceptors underlying behavioral performance revealed a nominal improvement in visual performance up to 4 weeks of age, with extensive changes in behavioral performance observed over the ages 5 weeks to 12.5 months after visual performance has become asymptotic (Kiorpes et al., 2003). Additionally, changes in the response properties of V1 neurons early in development contribute minimally to the improvement of visual sensitivity that takes place from 5 weeks until 24 months. Therefore, although the maturation of RF properties in V1 contributes to the behavioral improvement in visual function, other immaturities in extrastriate cortical circuits are likely to place limitations on visual performance.

#### Differential Development of Basic and Complex Neuronal Response Properties

Generally, complex neuronal response properties in higher order visual areas require a relatively protracted period of maturation in comparison to basic response properties in lower order visual areas which mature early in life and require less time to mature. For instance, in the ferret, basic RF properties such as orientation selectivity of cells in area 17 are immature at birth, but improve in the days after eye-opening and become adultlike at 42 days postnatal (Chapman and Stryker, 1993; McKay et al., 2001; Usrey et al., 2003). Similarly, in macaque V1, cells sensitive to a basic RF property such as binocular disparity are found immediately after birth, several weeks before the onset of stereopsis with spatial frequency response and response amplitude gradually developing over the first 4 weeks of age (Chino et al., 1997). Additionally, previous findings in macaques have shown the presence of adult-like RF center and surround interactions in V1 as early as postnatal day 14, however, RF surrounds of V2 neurons were not detectable until 4–8 weeks of age, and the interactions between RF center and surround in V2 neurons were immature (Kiorpes et al., 2003; Zhang et al., 2005). This suggests that neural circuits underlying the RF centers and surrounds undergo refinement at different rates. Differences in the rate of maturation of RF properties are more evident as we proceed toward more complex functions that are processed in higher order visual areas. A recent study discussing the development of selectivity to visual texture between areas V2 and V4 in macaque monkeys demonstrated the existence of a stronger response to higher order features of texture perception in V4 region, suggesting a gradual maturation of RF features in a hierarchical fashion (Okazawa et al., 2016). Interestingly, in ferret area 17, complex RF properties such as cross-orientation suppression and surround suppression are both present early in development, at the time of eye opening and change nominally with visual experience. Thus, it seems that although basic response properties of neurons often develop and mature before complex neuronal response properties, there are exceptions to this rule that depend to a large extent on the species. Furthermore, RF tuning properties of a neuron in primates and carnivores have been shown to correlate with the local structure of functional maps. For instance, neurons in primary visual cortex that lie in iso-orientation domains essentially have homogenous orientation preference, however, neurons that lie in pinwheel centers have a variety of preferences (Nauhaus et al., 2008). The location of a neuron within the orientation map greatly influences the fine tuning of orientation bandwidth such that fine-tuned cells are mostly found in regions of high homogeneity, whereas regions of rapid orientation change have cells corresponding to broader tuning. Given that the developmental timing and onset of local map structure differs between basic and complex response properties, both within and across species, it is possible then that differences in the local map structure could alter the rate of refinement of RF properties. This in turn could explain known differences in the rate of development of RF properties among species.

#### Role of Visual Experience and Effect of Deprivation During the Development of Neuronal Response Properties

An important question in developmental neuroscience is how early sensory experience contributes to the functional development and maturation of an adult brain. Visual experience differentially affects neuronal response properties, even among different species. Direct dependence of brain regions and their underlying mechanisms on visual experience may contribute to the observed differences in RF maturation among species. For example, spatial frequency tuning increases independently of visual experience in cats during the first 3 weeks of life, but is required for further improvement (Derrington and Fuchs, 1981). Direction selectivity in area 17 necessitates visual experience for even basic development in ferrets but not mice (Li et al., 2006; Rochefort et al., 2011). Cross-orientation suppression and surround suppression are present at eye opening in ferrets, thus suggesting that visual experience is not necessary for their emergence but is necessary for sustaining these functions (Popović et al., 2018). In preterm monkeys with light exposure, synapse formation proceeds normally, but for further fine tuning of RF properties visual experience is necessary (Bourgeois et al., 1989; Yuste et al., 1992; Anderson et al., 1993; Bourgeois and Rakic, 1996; Callaway, 1998; DeAngelis et al., 1999). Equally important is the role of experience in the form of a light-evoked signal or spontaneously generated neural signal in the overall development of the visual system (Ruthazer and Aizenman, 2010). Developmental events in the early phase of life depend on spontaneous neuronal activity before the period of eye opening and in certain instances rely on light passing through unopened eyelids (Krug et al., 2001; Colonnese et al., 2010; Xu et al., 2011; Ackman et al., 2012). A recent innovative study in ferrets demonstrated that visual development progresses through neural activity occurring before and at eye opening (Roy et al., 2020). Visual stimulation through closed and opening eyelids serves in shaping the neuronal response properties and provides instructive signals for cortical circuit formation (Roy et al., 2020). Collectively, these data suggest that differences exist in the type of experience or duration that differs among species, but even within a species among different response properties (Aboitiz et al., 2018). Nonetheless, little effort has been made to relate these differences to the behavioral ecology of a species, which is likely to play a major role in how visual experience differentially affects the refinement of neuronal response properties (Pallas, 2017).

Earlier studies in cats discussing the effect of light on the maturation of RF properties of cells in area 17 have shown that cells in dark reared animals were immature for orientation selectivity and direction preference stimuli (Blakemore and Van Sluyters, 1975; Buisseret and Imbert, 1976; Bonds, 1979). A comparison of the RF of neurons in the visual cortex of normal animals with those of dark-reared animals showed a significant increase in the number of orientation selective neurons in both normal and dark reared animals from 3 to 5 weeks of age. However, this is followed by a decrease in the number of orientation selective cells in dark reared animals after 5 weeks of age (Buisseret and Imbert, 1976). This suggests that the RF properties of orientation selective cells are present in both normal and dark reared cats until about 3,4 weeks of age (Sherk and Stryker, 1976), with the orientation map undergoing developmental changes in dark reared animals up to 3 weeks of age followed by subsequent degeneration after 5 weeks. This demonstrates that experience is necessary for the maintenance of responsiveness and selectivity of neurons (Crair et al., 1998). Therefore, it appears that differential maturation of RFs in the visual cortex may arise from both experience-independent mechanisms that have differentially evolved in various species, or through a channel of sensory experience in the early stages of life. Spontaneous neural activity underlying the sequence of developmental events in the early phase of life shapes the initial emergence of lower order visual functions, but more importantly orchestrates the onset and maintenance of higher form perceptions via permissive and instructive processes.

#### Maturation of Visual Function

Visual function at birth is relatively poor in human and non-human primates and gradually matures in the following months or years. Behavioral assessment of visual function in infants reveals that receptive field properties of neurons appear more developed than a behaviorally elicited response (Kiorpes et al., 2003; Kiorpes and Movshon, 2004b). The first study focused on the development of visual function was in infants looking at four different patterns; shift, search, compensation and focal patterns (Tronick and Clanton, 1971), however, the initial ground work in this field leading to the observation of infant’s basic perceptual abilities, preferential looking and visual discriminative abilities was already achieved in the fifties (Fantz, 1958, 1961, 1964). Extensive research has been carried out on the behavioral development of visual functions in humans (Teller, 1984, 1997; Braddick et al., 1986; Slater et al., 1988; Atkinson and Braddick, 1992; Parrish et al., 2005; Wattam-Bell et al., 2010; Braddick and Atkinson, 2011) and to a lesser extent in non-human primates (Boothe et al., 1988; Kiorpes et al., 2003; Kiorpes and Movshon, 2004a, b; El-Shamayleh et al., 2010). Given that behavioral development is largely studied in humans and non-human primates, this section focuses on well-studied basic functions such as orientation and direction preference, spatial and temporal contrast sensitivity, and visual acuity in these species. Likewise, there are ample data documenting developmental changes in complex functions such as global motion sensitivity, and contour integration perception in these species.

#### Developmental Timeline of Visual Functions Among Species

**FIGURE 2:**
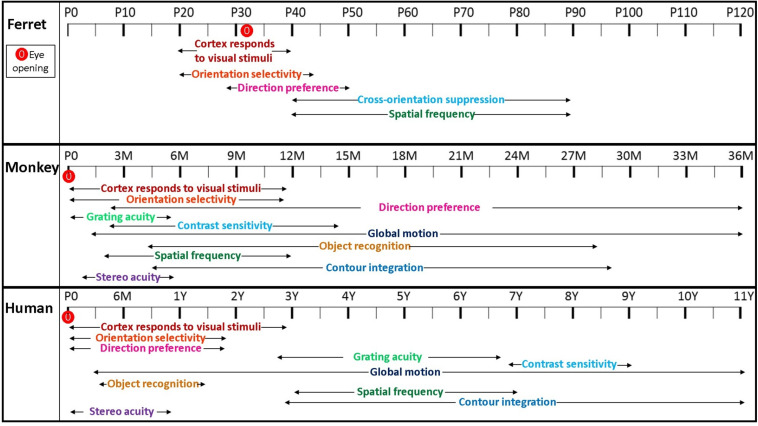
Timecourse illustrating visual function development in the ferret, monkey and human. A schematic illustrating the developmental timing of different visual functions reflecting relative onset and duration of maturation in different species. Eye opening in ferrets is around P30, and at birth in monkeys and humans. Cortex responds to visual stimuli as early as P20 in ferret whereas it is present at birth in both monkeys and humans. Unlike in both monkeys and humans, development of direction preference requires visual experience in the ferret. Orientation selectivity in ferret is present at the onset of visual experience by P20 and is fully mature by P45. Monkeys can detect motion direction discrimination responses as early as 3–5 weeks postnatally and these complex form cues continue to mature up to 3 years of age. In ferrets and humans, spatial frequency appears at a later stage of postnatal development, whereas, it is present at a relatively earlier timepoint of about 2 months of age in the monkey.

Figure 2 is a schematic we constructed to summarize the timecourse of visual function development in the ferret, monkey and human, based on physiological and behavioral data. The developmental timeline of different visual functions along with their period of maturation varies significantly among visual cortical areas and species (Sur and Leamey, 2001; Katz and Crowley, 2002; Hooks and Chen, 2007; Khalil and Levitt, 2014; Empie et al., 2015; Atkinson, 2017; Khalil et al., 2018). As shown in Figure 2, higher form perception such as contour integration develops and matures substantially later than relatively simple functions such as spatial acuity and contrast sensitivity in both human and non-human primates (El-Shamayleh et al., 2010). In humans, contour integration is undetectable in children younger than 3 years of age (Kovács et al., 1999), and in monkeys, the ability to perform a contour integration task develops late as well (Kiorpes and Bassin, 2003). Various aspects of visual perception develop at different rates and behavioral sensitivity to various perceptual inputs throughout development is an indication of time course-dependent refinement of these mechanisms. Neural limitations imposed on the visual system during development may include anatomical and physiological factors that are not fully mature and are important in the regulation of sensory information. The refinement of a neural circuit in a visual pathway can influence the rate of maturation of different visual functions, establishing the important relationship between behavioral and neuronal phenomena (Teller, 1984).

#### Differential Rates of Maturation of Basic and Higher Order Visual Functions

In humans, the progressive enhancement of visual functions such as visual acuity, orientation preference, contrast sensitivity, and motion detection is necessary for the development of higher order visual functions that require complex cognitive processing. Interestingly, none of these basic visual functions attain adultlike characteristics at the initial stages of development (Siu and Murphy, 2018). For instance, in humans, although orientation-selective cortical responses start to develop in early infancy, first appearing around 6 weeks of age (Braddick et al., 1986), mature orientation discrimination abilities require several months of visual experience before reaching adult levels. Likewise, the development of visual acuity in the macaque monkey requires 9 months after birth to fully mature, which in humans it takes approximately 5 years to reach similar adult levels (Teller, 1997). Physiological evidence suggests that spatial contrast sensitivity in macaque monkeys is fairly mature in newborns compared to a behaviorally elicited response (Kiorpes et al., 2003). Fundamental characteristics of spatial vision are established early in life and infant monkeys can detect gratings soon after birth, reaching mature levels of acuity and contrast sensitivity by the time they are one year of age (Boothe et al., 1988; Kiorpes, 1992). Given that many aspects of visual circuits in V1 attain adultlike characteristics in the first 8 weeks of life suggests that full maturation of RF properties partially accounts for the behavioral development (Kiorpes and Movshon, 2004a; Movshon et al., 2005). One caveat is feedback circuits in monkeys that continues to reorganize and refine up to 4 months of age (Kennedy and Burkhalter, 2004).

**FIGURE 3:**
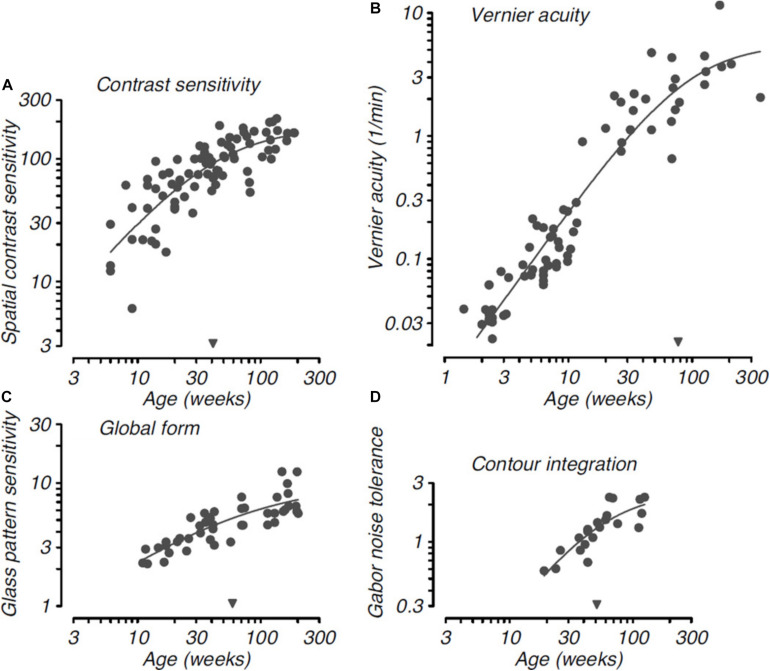
Developmental timecourse for basic and complex visual functions in monkeys. Basic visual functions: **(A)**. Spatial contrast sensitivity: **(B)**. Vernier acuity; and complex visual functions: **(C)**. Development of sensitivity to global structure in concentric Glass patterns: **(D)**. Development of ability to link Gabor elements to form a coherent contour as a function of the density of background noise, shown as a function of age. Smooth curves in each panel are Naka-Rushton functions fit to each dataset; the inverted filled triangles along the abscissa indicate the semi-saturation point for the functions, which provide a quantitative metric for the relative maturation of each visual function. Note the relatively late maturation of Vernier acuity compared with the other spatial vision metrics. Reproduced from “Visual development in primates: Neural mechanisms and critical periods” L, Kiorpes (2015), Developmental Neurobiology, 75(10), page 11. Copyright 2015 by John Wiley and Sons.

Higher order visual functions involving more complex form cues exhibit delayed maturation, requiring longer postnatal time to become adult like. Figure 3 reproduced from Kiorpes (2015) is a comparative timecourse of the development of basic versus complex visual functions in monkeys. As shown in Figure 3, the onset of basic visual functions such as spatial contrast sensitivity and vernier acuity is near birth. In contrast, complex visual functions like global form discrimination and contour integration are not measurable until about 10–20 weeks after birth. Research has shown that some features of visual texture perception are associated with lower order visual functions, whereas others are linked to higher form perception. For example, juvenile monkeys in early visual development can access texture form cues, and this may be due to the fact that intermediate-level form vision develops in concert with basic spatial vision (El-Shamayleh et al., 2010). However, sensitivity to luminance-defined form was delayed in the maturation process compared to both texture- and contrast-defined forms (El-Shamayleh et al., 2010). Similarly, human studies have suggested that infants as early as 2–5 months of age were able to detect global texture patterns (Norcia et al., 2005; Arcand et al., 2007), and by 3 months of age were able to identify texture patterns characterized by differences in stimulus orientation, size and contrast (Atkinson and Braddick, 1992; Sireteanu and Rieth, 1992). Different aspects of form vision develop over different time courses, together with processes that rely on evaluating local image content developing prior to those requiring global connectivity of multiple visual elements across a greater spatial extent (El-Shamayleh et al., 2010). For example, monkeys are unable to detect global structure patterns before 12 weeks of age (Kiorpes and Movshon, 2003) and are not capable of identifying extended contours before 20 weeks of age (Kiorpes and Bassin, 2003). These complex form cues continue to mature over the first 18–24 months after birth (El-Shamayleh et al., 2010).

#### Development of Orientation Selectivity and Direction Preference

Orientation sensitivity is one of the first visual functions to appear after birth in humans, monkeys and ferrets. In humans, some orientation discrimination capabilities are present at birth and orientation-selective responses are first detected around 6 weeks of age (Braddick et al., 1986). However, if lower reversal frequencies are used, the response could be recorded as early as 3 weeks after birth (Atkinson and Braddick, 1992). The response dynamics of orientation-selective neurons depends on intracortical interactions that continues to develop at least over the first 6 months of postnatal life (Braddick and Atkinson, 2011). Despite the fact that orientation-specific cortical responses develop in early infancy, mature orientation discrimination abilities require several months before becoming adult like in nature (Atkinson et al., 1988; Slater et al., 1988).

Although studies focusing on the development of direction preference in cats and ferrets have mostly resulted in physiological data (Blakemore and Van Sluyters, 1975; Buisseret and Imbert, 1976; Weliky et al., 1996; Li et al., 2006; Van Hooser et al., 2012; Roy et al., 2018), behavioral development of direction preference has largely been studied in humans and non-human primates (Wattam-Bell, 1991, 1992, 1996; Hatta et al., 1998). In the macaque monkey, orientation selectivity is present at birth, but direction preference develops to adult levels over the first 4 weeks of postnatal life (Hatta et al., 1998). Similarly, orientation selectivity and direction preference in humans are already present at the time of birth with responses related to direction preference slightly lagging behind orientation selective responses (Wattam-Bell, 1991). In humans, preferential looking toward a random-dot field in opposite directions of motion revealed a relatively earlier onset of directional preference at around 7 weeks postnatal (Wattam-Bell, 1992, 1996). It appears that motion based sensitivity for direction develops relatively later than overall direction selectivity. Furthermore, it was shown that an infant’s initial responses to direction preference was limited to a very narrow range of low velocities, and this range increases throughout development for higher velocities (Wattam-Bell, 1992; Braddick and Atkinson, 2011). The increase in the range of high speed velocities might possibly reflect the concurrent refinement of intracortical circuitry needed to recognize these large displacements, as the spatial extent of these circuits increases throughout development. In contrast, the limited spatial extent of intracortical circuitry early on in development is sufficient to support low-speed velocity responses which rely on fine displacements. While there is no study examining the effects of impoverished visual experience such as dark-rearing on direction selective responses in primates, quantitative psychophysical analysis in humans have demonstrated that individuals with poor visual experience in both eyes tend to exhibit a higher threshold of motion detection compared to those who have experienced good vision in one or both eyes, suggesting that experience may be a crucial factor for direction selectivity in primates as in carnivores (Ellemberg et al., 2002).

#### Development of Spatial and Temporal Contrast Sensitivity

Spatial contrast sensitivity including peak contrast sensitivity and spatial resolution are poor in both humans and monkeys at birth. Assessment of spatial contrast sensitivity in monkeys has revealed rapid development during the first 10–20 weeks, followed by a gradual more protracted period of development to adult levels over the remainder of the year (Boothe et al., 1988). A similar event occurs in humans whereby infants aged 5–12 weeks show rapid improvement in contrast sensitivity between 5 weeks and 8–12 weeks (Atkinson et al., 1977; Banks and Salapatek, 1978). Additionally, both visual acuity and contrast sensitivity follow a similar developmental timeline in human and non-human primates, attaining adultlike characteristics between 3 and 7 years in humans, and between 9 and 12 months of age in monkeys (Boothe et al., 1988; Kiorpes, 1992; Teller, 1997; Ellemberg et al., 1999; Skoczenski and Norcia, 2002; Stavros and Kiorpes, 2008). However, visual acuity in humans is fully matured to adult levels between 5 to 15 years of age, while the development of contrast sensitivity lags behind and reaches adult levels between the ages of 8 to 19 years (Leat et al., 2009). Grating acuity in newborn monkeys was significantly more mature than vernier acuity despite the fact that vernier acuity has a faster rate of development and both these functions become adultlike at the same time, around 40 weeks of age (Kiorpes, 1992). In humans, grating acuity matures more gradually and attains adult values at about 4–6 years of age (Mayer and Dobson, 1982; Carkeet et al., 1997; Ellemberg et al., 1999; Skoczenski and Norcia, 2002).

Moreover, temporal contrast sensitivity appears to mature ahead of spatial contrast sensitivity in both human (Ellemberg et al., 1999), and non-human primates, when adult levels are reached by about 6 months of age (Stavros and Kiorpes, 2008). Previous studies in humans and monkeys have suggested that late developmental changes such as actual loss of synapses in the visual cortex and behavioral differences could significantly contribute to the difference in the rate of maturation of visual acuity and contrast sensitivity (Mayer and Dobson, 1982; Leat et al., 2009). The observed difference in the sequence of maturation of spatial and temporal contrast sensitivity suggests their development is subserved by different neural mechanisms. Young infant monkeys show reduced sensitivity for all temporal frequencies, but sensitivity to high and low frequencies developed at different rates. On the other hand, temporal contrast sensitivity showed no significant change with age (Stavros and Kiorpes, 2008). Differential development of the magnocellular and parvocellular pathways could contribute to this difference as the magnocellular pathway controls the detection of low spatial, high temporal frequencies, while the parvocellular pathways regulate the detection of high spatial, low temporal frequencies (Stavros and Kiorpes, 2008).

#### Development of Global Visual Functions

Global motion sensitivity, a complex visual function that depends on extrastriate cortical processing requires considerable postnatal time to mature. It is important to distinguish between low-level mechanisms that provide local motion signals from higher-level mechanisms that integrate motion information across space and time. For instance, young monkeys are sensitive to global motion as early as 3–5 weeks postnatally, however, the complete maturation of this visual function continues up to 3 years of age (Kiorpes and Movshon, 2004a). Similarly, the use of glass patterns and random dot kinematograms (RDK) to reveal global form and motion sensitivity in young monkeys has shown that sensitivity to coherent motion in RDKs was measurable earlier than sensitivity to glass patterns. However, adult performance on both tasks was reached at a similar age (Kiorpes et al., 2012). Interestingly, a recent behavioral study in ferrets has established a relationship between motion integration performance of neurons in area PSS and motion integration capacity, demonstrating that ferrets are capable of perceptual motion and form integration, known to be complex visual functions that are typically associated with higher-order visual areas such as MT in monkeys and PSS in ferrets (Dunn-Weiss et al., 2019). Therefore, the ferret is a suitable model system for visual psychophysics, and is amenable to experimental testing involving higher-level visual functions.

Behavioral studies in humans have similarly documented the protracted period of maturation of global visual functions. Global motion responses are more prevalent than global form responses in 4 month olds, although the activation patterns were different in infants compared to adults (Wattam-Bell et al., 2010), These findings are therefore in line with previous studies suggesting that dorsal stream function matures earlier than the ventral stream. Texture-defined form sensitivity continues to mature up to age 10,11 years (Parrish et al., 2005). However, there is no significant improvement in global motion sensitivity, as measured by coherence threshold, between age 3 years and adult (Parrish et al., 2005). These results suggest that certain aspects of both global motion and texture defined form develop at different times in children. Collectively, behavioral data on the development of sensitivity to form and motion suggests that motion sensitivity may appear and mature earlier in humans and non-human primates than form sensitivity. In addition, behavioral data revealing earlier maturation of motion sensitivity, presumably reflecting dorsal stream functioning, is consistent with anatomical data (Condé et al., 1996; Bourne and Rosa, 2006).

#### Development of Contour Integration Perception

The visual system’s ability to group discrete elements with similar orientation into a continuous contour that can be detected is known as contour integration (Field et al., 1993). Neural correlates of contour integration include horizontal connections and feedback connections (Taylor et al., 2014; Pak et al., 2020). Earlier studies based on computational network models proposed to understand illusory contour responses suggest that neurons in higher order visual areas such as V2 might pool orientation selective feedforward inputs from lower order areas like V1 (Mignard and Malpeli, 1991). Furthermore, activated neurons in higher order visual areas such as V2 can provide feature specific feedback signal to neurons in V1, facilitating contour completion by effectively modulating V1 targets via recurrent activity (Mignard and Malpeli, 1991). A recent study in monkeys demonstrated the crucial role of top-down feedback from higher order area V4 to lower order area V1, effectively modulating the strength of local connectivity in V1 during a contour detection task (Liang et al., 2017). Therefore, there appears to be a strong association between higher and lower order visual areas, effectively synchronizing feedforward and feedback interactions across V1 and extrastriate visual areas, thereby facilitating contour completion. Furthermore, it seems that feedback and lateral connections interact more closely to mediate grouping of elements necessary for contour perception.

Behavioral evidence supporting mechanisms of contour detection suggest that this function is present in the early stages of development (Field et al., 1993; Kovács et al., 1999; Hipp et al., 2014; Taylor et al., 2014), however, full maturation of this function requires years of visual experience. Kovács et al. (1999) reported that children younger than 3 years of age were unable to identify a coherent contour defined by a circular ring of gabor patches embedded in noise, and their ability to perform the task improved into their teenage years. The authors attribute this early inability to functional immaturities in terms of their spatial range and not so much to the lack of long-range spatial interactions. Furthermore, studies investigating visual segmentation of oriented textures by infants showed that significant differences were observed between 10 and 16 weeks of age (Atkinson and Braddick, 1992). Differential responsiveness to random versus organized textures emerges no later than 2–5 months of age, and responsiveness to orientation-defined contours emerges around 6–13 months of age (Norcia et al., 2005). Other human studies revealed that development of sensitivity to global structure of glass patterns emerges around 6 years of age, however, these threshold patterns were found to be more adultlike at 9 years of age (Lewis et al., 2004; Palomares et al., 2012). In monkeys, the ability to perform a contour integration task develops late in postnatal life. Juvenile monkeys were first able to perform contour integration around 5–6 months of age when acuity development is complete and continues to mature well into the second postnatal year (Kiorpes and Bassin, 2003). Young monkeys were shown to discriminate the orientation of texture defined form around 6 weeks postnatal, and their behavioral sensitivity showed adultlike characteristics around 40 weeks of age (El-Shamayleh et al., 2010). Collectively, these studies suggest that although some aspects of contour integration perception emerge early in life both in humans and non-human primates, the full maturation of this complex visual functions requires a protracted period of development.

Full maturation of contour integration abilities results from developmental remodeling of cortical circuits that mediate contour detection and integration. Analysis of human brains ranging from 24 weeks of gestation to 5 years of age has revealed that although the basic anatomical structure of V1 is seen very early in development, however, anatomical features of vertical and horizontal connections in the visual cortex continue to refine throughout development (Burkhalter et al., 1993). Horizontal connections are actively involved in linking columns with collinear orientations suggesting that considerable development of contour detection capabilities that occur during the early postnatal period is due to the refinement of horizontal connections. This is primarily because horizontal connections are known to pool visual information over longer distances by effective integration of a greater area of visual space (Taylor et al., 2014).

## Conclusion

In the present review, we provided a comprehensive discussion on the development of visual cortical circuits and function in three mammalian species (ferret, monkey, and human), in an effort to highlight the importance of comparative analysis of the differential rates of development. This is immediately relevant to our understanding of human visual perception as it may help reveal common mechanisms necessary for healthy visual development during infancy and adulthood. Although a large volume of experimental data suggests that basic RF properties and visual functions across species mature earlier than complex ones, few studies have focused on revealing mechanisms that can explain the differential rate of development among species, and how response properties evolved to serve species-specific adaptive behavior. It is clear that rudimentary RFs are generated early in life through interactions between molecular cues and spontaneous activity, while visual experience fine tunes circuits to improve the selectivity of neuronal response properties. However, further studies are needed to establish why visual experience differentially affects the development of neuronal response properties and functions among species. This will likely require an integrated view of visual system development and evolution of adaptive behavior in mammals.

## Author’s note

Permission must be obtained for use of copyrighted material.

## Permission to Reuse and Copyright

Permission to adapt Figure 1 from Sur and Leamey (2001) was obtained from the licensed content publisher Springer Nature with license number 4753540300780.

Permission to reproduce Figure 3 from Kiorpes, 2015 was obtained from the licensed content publisher, John Wiley and Sons with license number 4750830907023.

## Author Contributions

CD and RK contributed to writing and editing of the manuscript. RK contributed to the conception and organization of the manuscript. CD and RK approved the final manuscript.

## Conflict of Interest

The authors declare that the research was conducted in the absence of any commercial or financial relationships that could be construed as a potential conflict of interest.
